# From Hepatitis to Encephalitis: Neuroinvasion and Antiviral Development in Rift Valley Fever Virus Infection

**DOI:** 10.3390/pathogens15090891

**Published:** 2026-08-25

**Authors:** Sawrab Roy, Lei Shi, Shuhui Liu, Wenjun Ma

**Affiliations:** 1 Department of Pathobiology and Integrative Biomedical Sciences, College of Veterinary Medicine, University of Missouri, Columbia, MO 65211, USA; 2 Department of Molecular Microbiology and Immunology, School of Medicine, University of Missouri, Columbia, MO 65211, USA; 3 NextGen Center for Influenza and Emerging Infectious Diseases, University of Missouri, Columbia, MO 65211, USA

**Keywords:** Rift Valley fever virus, neuropathogenesis, antiviral treatment, blood–brain barrier penetration, therapeutic window, combination therapy

## Abstract

Rift Valley fever virus (RVFV) is a mosquito-borne zoonotic pathogen that causes substantial livestock losses and a range of severe human illnesses, including hemorrhagic disease, hepatitis, retinitis, vision loss, and delayed encephalitis. Despite its public health and One Health importance, no approved RVFV-specific antiviral therapy or licensed human vaccine is available. Therapeutic development is challenged by the progression of RVFV disease from acute viremia and hepatic injury to delayed neurologic and ocular complications, highlighting the need for countermeasures that protect both systemic organs and CNS tissues. This review examines RVFV antiviral development in the context of neuroinvasive disease. We summarize evidence on RVFV neuroinvasion and central nervous system injury, including route-dependent entry, blood–brain barrier interactions, immune responses, neuroinflammation, and neuronal damage. We then evaluate major antiviral strategies by mechanism, treatment timing, tissue exposure, and central nervous system relevance. Finally, we propose that future RVFV therapeutics are assessed not only by survival, viremia, and hepatic viral-load endpoints, but also by blood–brain barrier penetration, brain pharmacokinetics, efficacy in neuroinvasive models, delayed-treatment activity, and protection against encephalitis-associated injury. This framework may help prioritize antivirals that control both acute systemic disease and delayed neurological complications.

## 1. Introduction

Rift Valley fever virus (RVFV) remains a major One Health threat nearly a century after its first identification during a sheep epizootic in Kenya’s Rift Valley in 1931 [1,2]. As a climate-sensitive and geographically expanding zoonotic pathogen, RVFV continues to cause recurrent outbreaks across endemic regions, including 67 documented Rift Valley fever outbreak events in East Africa between 2010 and 2024 and a 2025 outbreak in Mauritania and Senegal with 404 confirmed human cases and 42 deaths [3,4,5]. Reflecting its epidemic potential, agricultural impact, occupational risk, and biodefense relevance, RVFV is prioritized by World Health Organization (WHO) for medical-countermeasure development and designated by the U.S. Federal Select Agent Program as an overlap select agent [6,7].

RVFV is maintained predominantly by mosquito-borne transmission and causes severe epizootics in domestic ruminants, including abortion storms and high neonatal mortality. Humans are infected through mosquito bites, contact with infected animal tissues, exposure to blood or body fluids, and potentially through aerosols generated during slaughtering, necropsy, or laboratory manipulation [2,5,8,9]. Although human Rift Valley fever is often self-limited, severe disease can include hepatitis, hemorrhagic fever, retinitis, and delayed-onset encephalitis [10,11,12]. Neurologic disease, first recognized during the 1974–1976 South Africa outbreak [13] has since emerged as a critical but incompletely understood manifestation. During the Saudi Arabian epidemic, neurologic manifestations were reported in 17.1% of cases, and subsequent analyses estimated a 53% case-fatality rate among patients with neurologic involvement [14,15]. Meningoencephalitis was also reported as a late complication among severe hospitalized cases from Gizan, Saudi Arabia, where 56 of 165 patients died [16]. These observations underscore the disproportionate clinical impact of RVFV-associated central nervous system (CNS) disease and the need to define the mechanisms governing RVFV neuroinvasion, CNS tropism, and neuropathogenesis.

Despite this burden, no RVFV-specific antiviral therapy or licensed human vaccine is available, and clinical care remains largely supportive, although veterinary vaccines are used in livestock in some endemic settings [2,17,18]. Antiviral discovery has identified multiple candidates and targets, from early ribavirin, liposomal ribavirin, poly(ICLC), and interferon-based studies to more recent approaches targeting the L polymerase, endonuclease domain, viral entry and fusion, and nucleoprotein–RNA interactions [19,20,21,22,23,24,25,26,27,28,29,30,31,32]. However, translation has been constrained by narrow treatment windows, toxicity, uncertain pharmacokinetics, incomplete validation against pathogenic strains, and limited testing in neuroinvasive disease models. A key gap is whether antivirals that reduce acute systemic or hepatic disease can also prevent delayed CNS seeding and encephalitis.

This review integrates current knowledge of RVFV neuroinvasion and neuropathogenesis with advances in antiviral development. We synthesize evidence for progression from systemic infection to CNS involvement, including route-dependent neuroinvasion, blood–brain barrier (BBB) traversal, neuroinflammatory responses, and neuronal injury. We then evaluate direct-acting, host-directed, immune-modulating, and combination antiviral strategies, emphasizing therapeutic windows and CNS-relevant endpoints needed to control both acute hepatic disease and delayed neuroinvasive complications.

## 2. Replication Cycle of RVFV

RVFV is an enveloped, tri-segmented RNA virus classified within the genus *Phlebovirus* in the family *Phenuiviridae*. Its genome comprises large (L), medium (M), and small (S) RNA segments of negative-sense or ambisense polarity, and each segment encodes functions that represent potential points of antiviral intervention. The L segment encodes the viral RNA-dependent RNA polymerase, which catalyzes genome transcription and replication, including cap-snatching-dependent initiation of viral mRNA synthesis; accordingly, the polymerase and endonuclease activities are major targets for direct-acting antivirals. The M segment encodes a polyprotein that is proteolytically processed to generate envelope glycoproteins Gn and Gc, which mediate receptor engagement, endosomal uptake, and low-pH-triggered membrane fusion, as well as the nonstructural NSm/78 kDa proteins that contribute to viral fitness and modulation of host–cell responses. These early entry and fusion steps create opportunities to block viral attachment, endosomal trafficking, or membrane fusion before productive infection is established. The S segment employs an ambisense coding strategy to encode the nucleocapsid protein N, which encapsidates viral RNA to form ribonucleoprotein complexes, and NSs, a principal virulence factor that antagonizes host innate immune responses, making immune antagonism and ribonucleoprotein formation additional therapeutic targets. RVFV attachment and entry appear to involve multiple host–cell factors rather than a single universal receptor, including heparan sulfate, C-type lectins such as DC-SIGN/L-SIGN, non-muscle myosin heavy chain IIA, and the recently identified host entry factor LRP1 [33,34]. Following attachment and endocytosis, endosomal acidification triggers Gn/Gc-mediated fusion and cytoplasmic release of viral ribonucleoproteins, enabling primary transcription, viral protein synthesis, antigenome production, and genome replication. Newly synthesized ribonucleoproteins are then recruited to Golgi membranes through interactions with viral glycoproteins, facilitating virion assembly and budding, followed by egress of mature particles through the secretory pathway [12,35,36,37,38]. Thus, the RVFV replication cycle provides multiple mechanistically distinct antiviral entry points, spanning attachment and fusion, polymerase and endonuclease function, ribonucleoprotein assembly, innate immune antagonism, virion morphogenesis, and egress.

## 3. Clinical and Experimental Perspectives on the Biphasic Progression of Rift Valley Fever Virus Disease

**Figure 1:**
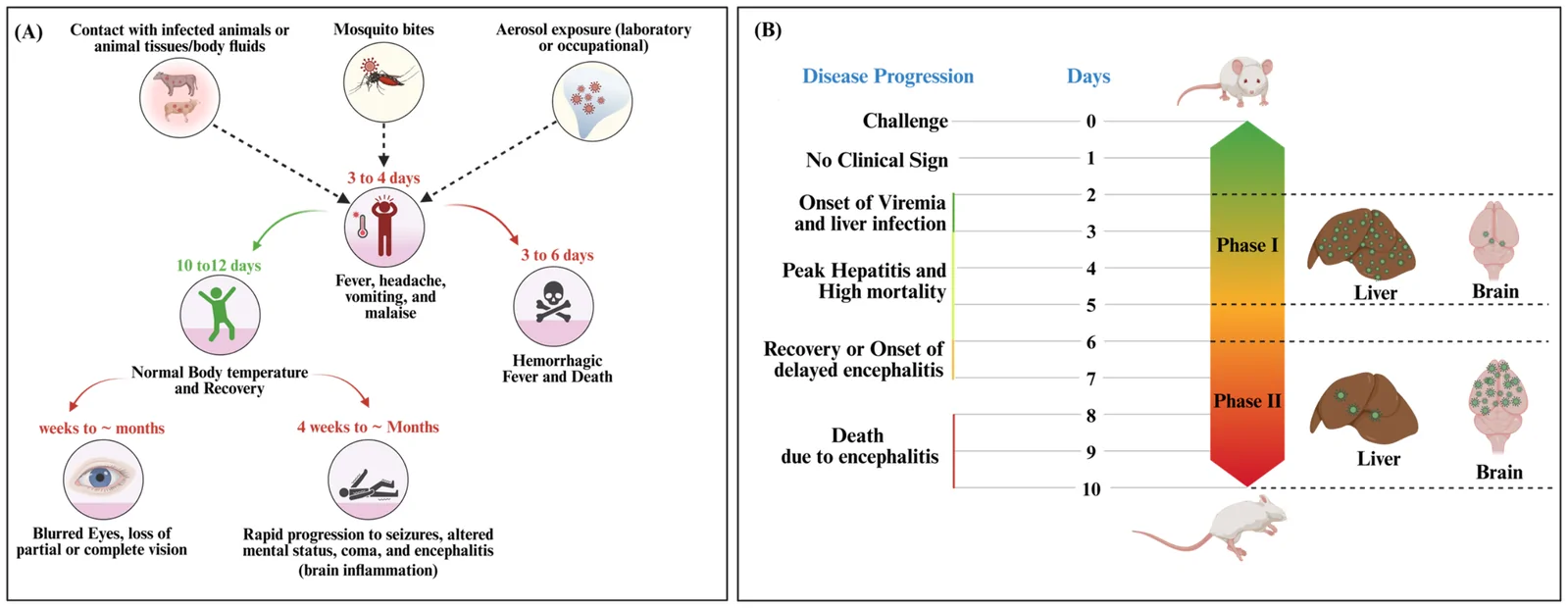
Clinical and experimental rationale for biphasic RVFV disease. (**A**) Human RVFV infection can progress from an early acute viremic and hepatotropic phase to delayed neuro-ocular complications in a subset of severe cases. (**B**) The BALB/c mouse model recapitulates the biphasic course of RVFV disease, with an early liver-associated phase followed by delayed CNS involvement [41]. Mice infected with virulent RVFV typically show no obvious clinical signs at 1 day post infection (dpi). By 2 dpi, viremia and hepatic infection become detectable. During Phase I, severe hepatitis and high mortality occur between 3 and 6 dpi. Surviving mice generally clear virus from the liver and blood by 7 dpi; however, delayed encephalitis emerges thereafter, leading to mortality during Phase II, typically between 8 and 10 dpi. Created in BioRender. Roy, S. (2026) https://BioRender.com/.

A central feature of Rift Valley fever is its biphasic pathogenesis, in which an early viremic or hepatotropic phase can be followed by delayed ocular or neurological disease. In humans, RVFV infection typically presents as an acute febrile illness, but severe cases may progress to hepatitis, hemorrhagic disease, retinitis or vision loss, and meningoencephalitis (Figure 1A) [11]. This delayed onset of retinitis and encephalitis after resolution of fever suggests that neuro-ocular disease is not simply an extension of acute systemic infection, but reflects a temporally distinct phase of virus dissemination, tissue invasion, and local injury [11,12]. Experimental evidence reinforces this two-stage framework. In mice, early high-titer viremia, hepatocyte infection, apoptosis, and severe hepatitis dominate acute disease, whereas animals that survive or resist the acute hepatic injury may subsequently develop CNS infection and encephalitis (Figure 1B) [39,40,41]. Nonhuman-primate studies provide translational support, demonstrating severe systemic disease and fatal encephalitis in susceptible models, particularly after aerosol exposure [42,43,44]. Together, clinical, rodent, and nonhuman primate data support a model in which RVFV first amplifies systemically and in the liver, then—depending on exposure route, host susceptibility, immune control, and tissue barriers—seeds immune-privileged neuro-ocular sites. This temporal dissociation raises a fundamental mechanistic question: how does RVFV transition from acute systemic and hepatic replication to delayed CNS pathology after apparent clinical recovery? Current evidence points to overlapping mechanisms, including route-dependent neural access, BBB interaction, inflammatory cell trafficking, local viral replication, neuroinflammation, and direct neuronal injury. Integrating these mechanisms is essential because therapeutic success during the acute phase may not be sufficient once virus has entered or persisted within the CNS. Therefore, RVFV antiviral strategies should be assessed not only for their ability to reduce viremia and liver disease, but also for their impact on CNS-relevant outcomes, including CNS seeding, brain viral burden, neurologic signs, neuro-ocular injury, and direct measurement of drug exposure in the brain or cerebrospinal fluids.

## 4. Neuropathogenesis of RVFV Infection

RVFV neuroinvasion is highly route-dependent and likely arises from convergent mechanisms that include olfactory–cribriform spread after inhalational exposure, hematogenous access to the neurovascular unit, direct BBB crossing, possible leukocyte-associated trafficking, local CNS replication, neuroinflammation, and neuronal injury (Table 1).

### 4.1. Exposure Route Shapes Neuroinvasion Pathways

#### 4.1.1. Aerosol and Intranasal Exposure: Olfactory–Cribriform Entry

**Figure 2:**
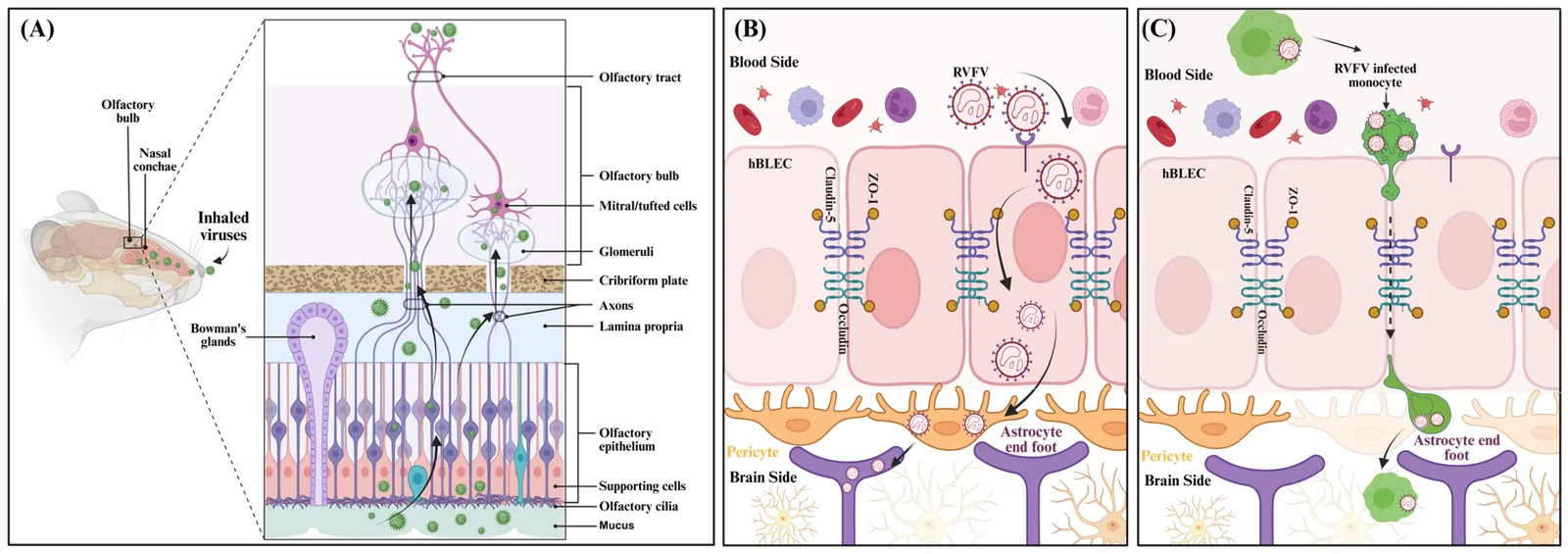
Route-dependent neuroinvasion and neuropathogenesis of RVFV infection. (**A**) Inhalational exposure may favor olfactory epithelial infection and transneural spread through the cribriform plate into the olfactory bulb. (**B**) Peripheral infection may promote systemic replication and viremia, enabling RVFV interaction with the neurovascular unit and direct BBB crossing. (**C**) RVFV may also exploit inflammatory cell trafficking as a proposed “Trojan horse” mechanism during BBB disruption and neuroinflammation. hBLEC; human brain-like endothelial cells. Created in BioRender. Roy, S. (2026) https://BioRender.com/.

Respiratory exposure models provide the clearest evidence for a defined route of RVFV neuroinvasion. In mice, aerosol exposure produced earlier and more severe neuropathology than subcutaneous exposure, even though both routes caused acute hepatitis. In addition, ribavirin was ineffective after aerosol exposure but partially protective after subcutaneous challenge [46]. These findings indicate that respiratory exposure accelerates CNS invasion and sharply compresses the therapeutic window for antiviral intervention. In a rat aerosol model, Boyles et al. detected RVFV-infected cells in the olfactory epithelium, across the cribriform plate, and within the olfactory bulb glomerular layer within two days of infection [45], supporting an olfactory-cribriform bulb route in which inhaled virus reaches the olfactory bulb directly from the nasal mucosa (Figure 2A). This pathway is particularly relevant to laboratories, abattoir, veterinary, and livestock-handling exposures involving infectious aerosols or respiratory-mucosal contact and likely contributes to the rapid brain involvement and severe encephalitis observed in aerosol models. Because it may not fully explain mosquito-borne disease; however, olfactory–cribriform entry should be considered as an exposure-specific dominant mechanism rather than a universal route of RVFV neuroinvasion. 

#### 4.1.2. Peripheral or Mosquito-Borne Infection: Hematogenous-Neurovascular Entry

Following peripheral or mosquito-borne infection, hematogenous dissemination to the neurovascular unit is the most plausible mechanism of CNS seeding. The blood–brain barrier (BBB), primarily formed by specialized brain endothelial cells supported by pericytes, astrocytic end-feet, basement membranes and microglia. Although BBB limits paracellular diffusion, viruses can exploit it through endothelial infection, transcytosis, barrier leakage, or infected leukocyte trafficking [54]. In an in vitro BBB model, Quellec et al. showed that RVFV crosses from the blood-facing side by sequentially infecting brain endothelial cells, pericytes and astrocytes without detectable tight-junction disruption or loss of barrier function (Figure 2B) [47]. Thus, RVFV can access CNS-associated cells without overt BBB breakdown, consistent with early CNS seeding during viremia. Walters and colleagues likewise reported that increased vascular permeability developed during the later stages of aerosol-induced RVFV infection in Lewis rats and coincided temporally with increased matrix metalloproteinase-9 (MMP-9) [48]. Because RVFV replication in the brain was detectable several days before the onset of vascular leakage, BBB permeability is unlikely to represent the initial route of neuroinvasion. Instead, ongoing CNS infection may induce inflammatory mediators, matrix metalloproteinases, leukocyte recruitment, endothelial activation, and glial responses that progressively compromise neurovascular integrity and exacerbate tissue injury. Thus, BBB permeability is more appropriately viewed as a secondary, disease-amplifying consequence of established CNS infection rather than the primary gateway for viral entry into the brain.

A Trojan-horse mechanism refers to viral entry into the CNS within infected leukocytes, particularly monocytes or macrophages that transmigrate across the BBB [54]. Leukocyte-associated “Trojan-horse” entry remains biologically plausible but insufficiently demonstrated for RVFV. Although RVFV encephalitis is accompanied by CNS infiltration of macrophages and neutrophils, and myeloid cells may contribute to inflammation or viral dissemination (Figure 2C), their accumulation does not establish that infected leukocytes initiate CNS entry [49]. These cells may instead be recruited after CNS infection through chemokine signaling, vascular activation, and increased BBB permeability. Current RVFV-specific evidence therefore supports a hierarchy of neuroinvasion mechanisms: olfactory–cribriform entry predominates after inhalational exposure, whereas direct neurovascular crossing is most relevant during hematogenous infection; Trojan-horse trafficking may function as an accessory pathway under inflammatory conditions. This hierarchy has direct therapeutic implications. Antivirals active during early viremia may reduce endothelial infection and CNS seeding, whereas their effectiveness against established CNS infection will likely depend on brain exposure, antiviral potency in CNS-resident cells, and treatment timing. Therefore, high-priority RVFV antiviral candidates should be evaluated for BBB penetration, brain pharmacokinetics, delayed-treatment efficacy, and activity in neuroinvasive disease models.

### 4.2. Systemic Adaptive Immunity Limits Peripheral Replication and Prevents CNS Seeding

Within this route-dependent framework, adaptive immunity primarily determines whether systemic infection progresses to CNS seeding. In C57BL/6 mice infected with attenuated ΔNSs RVFV, B cells and CD4^+^ T cells, but not CD8^+^ cells, were required for efficient viral clearance and protection from neurological disease. CD4^+^ T-cell depletion compromised IgG and neutralizing antibody responses and increased susceptibility to neurologic disease, whereas intact CD4^+^ T-cell immunity promoted clearance of virus from peripheral tissues [50]. Route also shaped immune control: intranasal ΔNSs RVFV infection caused fatal encephalitis with delayed antibody responses, persistent peripheral infection, elevated CNS viral RNA, proinflammatory activation, and infiltration of activated T-cells, whereas footpad infection produced subclinical disease associated with rapid induction of antibody and T-cell responses [10]. Because T-cell depletion did not significantly alter survival after intranasal infection, lethal disease was unlikely primarily attributable to T-cell-mediated immunopathology [10]. These findings extend the neuroinvasion model above by showing that early humoral and CD4+ T-cell-dependent control limits the viremic window during which RVFV can engage the BBB, olfactory mucosa, or other CNS entry interfaces. This interpretation is reinforced by evidence that humoral immunity alone protects CC057/Unc mice from late-onset encephalitis after percutaneous challenge and that passive immune serum confers time- and dose-dependent protection [55]. Thus, prevention of RVFV encephalitis likely depends on early containment of peripheral replication before even limited viral seeding of the brain occurs, underscoring the importance of early neutralizing antibody responses for vaccines, passive immunotherapy, and antiviral intervention.

### 4.3. Brain-Intrinsic Immunity Balances Viral Control and Inflammatory Injury

If peripheral control fails and RVFV accesses the CNS through olfactory or neurovascular routes, brain-intrinsic immune pathways become central determinants of viral containment and tissue injury. Microglia, the resident macrophage-like immune cells, sense infection, produce interferons and chemokines, coordinate leukocyte recruitment, and support tissue repair. Hum and colleagues demonstrated that RVFV infects microglia and activates antiviral transcriptional programs through mitochondrial antiviral-signaling protein (MAVS), a RIG-I-like receptor, independently of TLR3 and TLR7 [51]. In vivo, MAVS deficiency increased brain viral burden and mortality, impaired microglial type I interferon and interferon-stimulated gene expression, and dysregulated lymphocyte infiltration [51]. These findings identify microglial MAVS signaling as a key CNS-intrinsic antiviral axis that acts after neuroinvasion has occurred. However, the same inflammatory programs that support viral control can amplify neuropathology once CNS infection is established. Lethal RVFV encephalitis is characterized by inflammatory cell infiltration and neuronal injury. In aerosol-infected Lewis rats, neutrophils and macrophages dominate the CNS infiltrates, although blockade of neutrophil migration alone does not fully rescue disease [49]. Ex vivo brain-slice systems and neuronal infection models reveal that RVFV infects CNS tissue, activates microglia and astrocytes, induces cytokine and chemokine expression, and engages regulated cell-death pathways including apoptosis, pyroptosis, and necroptosis [52,53]. Because mature neurons have limited regenerative capacity, even transient viral replication or inflammatory injury may produce persistent functional deficits when vulnerable circuits are affected. Together with the neuroinvasion mechanisms described above, these data argue that RVFV countermeasures must be timed and distributed to address two linked phases: prevention of CNS seeding during peripheral viremia and control of viral replication, neuroinflammation, and neuronal injury after CNS entry. CNS-directed therapeutic strategies should therefore aim to combine rapid antiviral activity with experimentally defined brain exposure, preservation of neuronal integrity, and control of injurious inflammation before irreversible neuropathology develops.

## 5. Advances in Antiviral Strategies Against Rift Valley Fever Virus

Antiviral development is typically organized around two complementary therapeutic paradigms: direct-acting agents that inhibit viral proteins or replication processes, and host-directed agents that target cellular factors required for viral replication or disease progression. Direct-acting antivirals block virus-encoded functions essential for productive infection, including receptor engagement, membrane fusion, genome replication and transcription, ribonucleoprotein assembly, or virion maturation and release. This strategy is clinically validated by multiple antiviral classes, including oseltamivir against influenza virus neuraminidase, sofosbuvir against the hepatitis C virus NS5B RNA-dependent RNA polymerase, and nirmatrelvir against the SARS-CoV-2 main protease [56,57,58,59]. In contrast, host-directed antivirals interfere with cellular receptors, trafficking pathways, metabolic programs, or signaling networks that viruses exploit for entry, replication, immune evasion, or pathogenesis. Clinically relevant examples include maraviroc, which blocks the CCR5 co-receptor used by CCR5-tropic HIV-1, and bulevirtide, which prevents NTCP-mediated HBV/HDV entry into hepatocytes [60,61]. This framework is useful for evaluating RVFV countermeasures because studies have implicated both viral targets, including the L polymerase, endonuclease domain, glycoprotein-mediated fusion, and nucleoprotein–RNA interactions, and host-associated pathways, including PI3K-Akt, NF-κB/IKK, proteasome activity, PP1, mTOR/p70S6K, and innate immune signaling. Here, we summarize antiviral agents and therapeutic strategies evaluated against pathogenic RVFV or the attenuated MP-12 strain, as depicted in Table 2.

### 5.1. Direct-Acting Antivirals Targeting the Viral Replication Machinery

**Figure 3:**
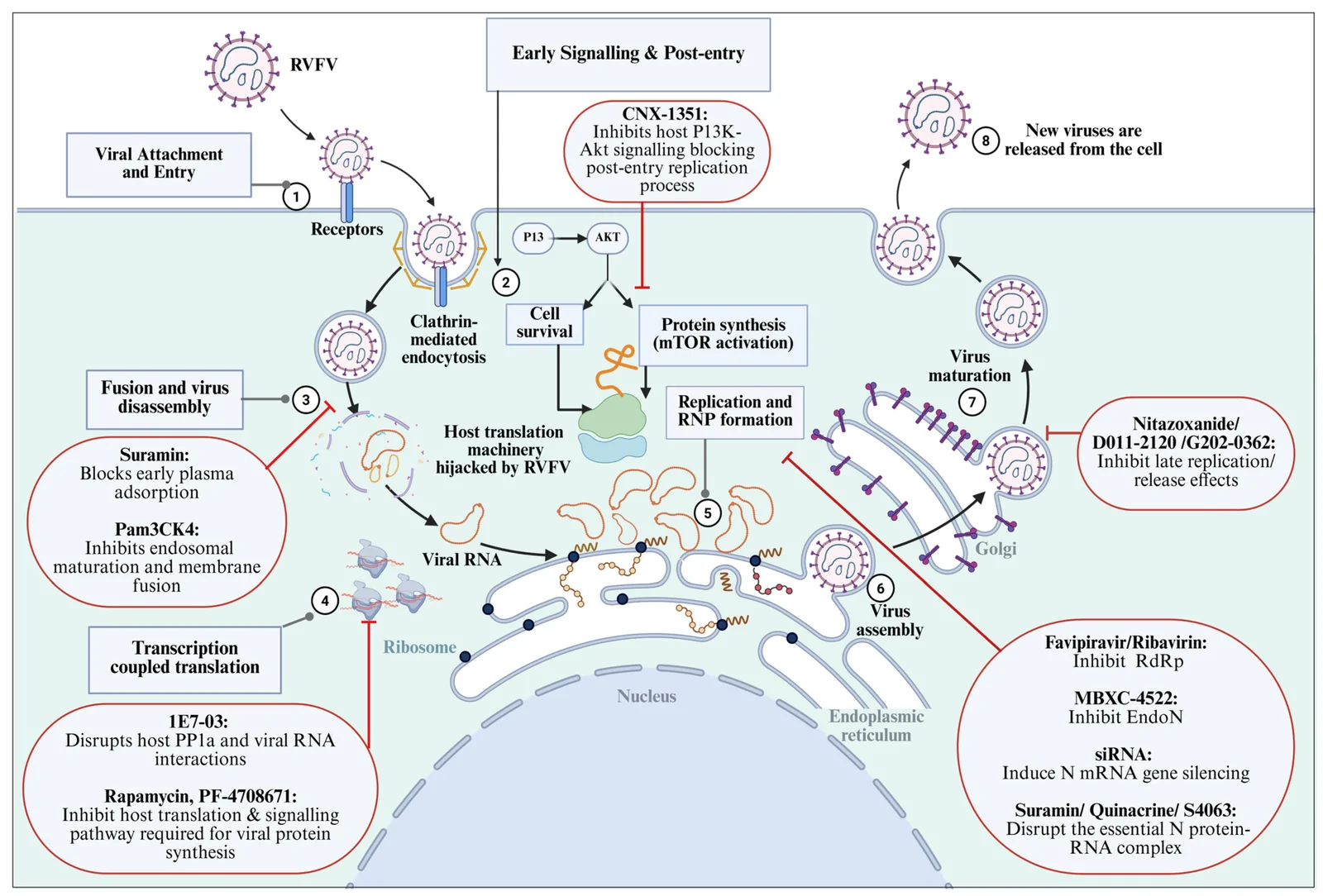
Replication cycle of RVFV infection and antiviral intervention points. RVFV first attaches to the host cell and enters through endocytosis (1–2). Following fusion and virus disassembly, the viral genome is released into the cytoplasm (3). Viral transcription and translation then produce viral proteins, followed by genome replication and RNP formation (4–5). Newly synthesized viral components are subsequently assembled into progeny virions (6), undergo maturation (7), and are released from the infected cell (8). Representative antiviral targets and intervention points are indicated throughout the replication cycle. Created in BioRender. Roy, S. (2026) https://BioRender.com/.

The RVFV replication cycle presents multiple points for antiviral intervention, including attachment, endocytosis, Gc-mediated fusion, cytoplasmic transcription and genome replication, nucleocapsid assembly, glycoprotein processing, Golgi-associated budding, and virion release (Figure 3). These steps provide the mechanistic foundation for direct-acting inhibitors, including fusion inhibitors, nucleoprotein–RNA inhibitors, nucleoside analogs, polymerase inhibitors, endonuclease inhibitors, and assembly or egress inhibitors. 

#### 5.1.1. Viral Entry and Gc-Mediated Fusion

Entry inhibition is attractive because it acts before genome amplification and may prevent downstream CNS seeding. RVFV Gc is a class II fusion protein that undergoes low-pH-triggered conformational rearrangement in endosomes to mediate membrane fusion. Peptides derived from the RVFV Gc stem region inhibited RVFV infectivity by blocking the fusion [27]. A single RVFV-derived peptide also inhibited unrelated enveloped RNA viruses with class I, class II, and class III fusion proteins, including Ebola virus, Andes virus, and vesicular stomatitis virus, suggesting membrane-associated fusion intermediates may possess conserved, targetable structural features shared across diverse viral families [27].

Evidence from neuroinvasive models further supports fusion as a CNS-relevant target. Griesman and colleagues screened a diverse library of innate immune ligands in neurons and identified the lipopeptide Pam3CSK4 as an inhibitor of RVFV and related bunyaviruses. Although classically recognized as a TLR2 ligand, Pam3CSK4 retained anti-RVFV activity in TLR2-deficient primary cortical neurons, suggesting that its antiviral effect does not strictly depend on canonical TLR2 signaling and may be linked, at least in part, to suppression of viral fusion. In a mouse encephalitis model, Pam3CSK4 protected against brain infection and mortality [28], positioning fusion blockade as a potential therapeutic strategy for established neurotropic infection and identify fusion inhibition as a CNS-relevant therapeutic strategy, rather than solely pre-exposure prophylaxis.

Suramin may also affect entry-related steps, although its best-characterized anti-RVFV activity is disruption of N–RNA interactions. Time-of-addition studies indicated that suramin can act at multiple stages, including early uptake or entry-associated events and later ribonucleoprotein-related processes [30]. Because entry inhibitors are most effective early, their translational value is likely greatest for high-risk exposure scenarios, laboratory accidents, livestock outbreak containment, or combination regimens aimed at reducing early viremia and limiting neuroinvasion.

#### 5.1.2. Early Post-Entry Signaling and Single-Cycle Screening Platforms

Because pathogenic RVFV requires high-containment handling, safer platforms are essential for antiviral discovery and mechanistic studies. Gao and colleagues generated stable replicon cells maintaining L and S segments and used glycoprotein trans-complementation to produce single-cycle viral replicon particles. This system recapitulates authentic entry and replication while reducing biosafety risk [62]. This system identified CNX-1351, a small-molecule antiviral with potent antiviral activity and limited cytotoxicity across multiple cell types. Time-of-addition, minigenome, and replicon assays indicated a predominantly post-entry effect on viral genome replication or replication-associated host processes, rather than attachment or internalization. Mechanistically, CNX-1351 is a covalent inhibitor of the PI3Kα isoform encoded by PIK3CA; transcriptomic profiling revealed broad perturbation of PI3K-Akt-linked pathways, and LY294002 produced a similar anti- RVFV phenotype [62]. Because no direct viral target has been identified, CNX-1351 is best viewed as a host-directed replication-stage inhibitor rather than a confirmed direct-acting polymerase inhibitor. Although replicon and reporter-virus systems cannot substitute for confirmation using pathogenic RVFV strains, they are useful for defining effects on viral entry, replication, and host signaling. These platforms also help prioritize candidates for infectious-virus validation, resistance analysis, pharmacokinetic studies, and evaluation in neuroinvasive disease models.

#### 5.1.3. Targeting the Nucleoprotein and Ribonucleoprotein Complex

Because high-affinity N–RNA interactions are required for RVFV ribonucleoprotein assembly and viral replication, the N–RNA interface represents a mechanistically defined direct-acting antiviral strategy [29,30]. Ellenbecker and colleagues used a fluorescence polarization-based high-throughput assay to screen 26,424 compounds for inhibitors of N–RNA complex formation, identifying leads from FDA-approved, drug-like, and natural-product libraries [29]. Suramin was subsequently shown to suppress RVFV replication in human cells by disrupting sequence-specific and nonspecific N–RNA interactions and dissociating high-molecular-mass RNP complexes [30]. Although limited by toxicity and unfavorable pharmacokinetics, suramin provides proof of concept that pharmacologic destabilization of RVFV RNPs can inhibit replication.

The essential and relatively conserved nature of N has also supported RNA interference–based strategies that suppress N-dependent RNP function by reducing viral RNA or protein expression. Scott et al. showed that Pol III-driven shRNAs targeting the RVFV S segment reduced NSs and N expression; N-directed shRNAs diminished nucleocapsid accumulation and RVFV replication-associated markers, whereas NSs-directed shRNAs reduced NSs-mediated host transcriptional suppression and cytotoxicity [71]. Faburay and Richt found that all four N-specific siRNAs significantly reduced viral protein expression and replication in MP-12-infected cells, whereas an L-targeting siRNA exhibited no detectable antiviral activity and additionally N-specific siRNA treatment also preserved PKR, consistent with reduced replication and attenuation of NSs-mediated innate immune antagonism [72]. Ahmed et al. further identified N-targeting siRNAs with prophylactic and post-infection activity, including D2, which nearly abolished N mRNA accumulation and markedly reduced N protein expression at 30 nM and pooled siRNAs also suppressed replication after viral infection [73].

Together, small-molecule and RNAi studies establish N as a high-priority antiviral target because it coordinates RNP assembly, genome replication, transcriptional competence, RNA stabilization, and genome packaging. The N-RNA interface offers a biochemical target for small-molecule disruption, whereas conserved N-coding sequences enable sequence-specific RNA-based inhibition. However, both approaches remain early in translation. Small-molecule N-RNA inhibitors require optimization for potency, selectivity, intracellular exposure, toxicity profiles, and pharmacokinetics, as reported inhibitors remain largely at biochemical-hit or early cell-validation stage [29,74]. RNAi-based countermeasures face additional delivery challenges, including nuclease susceptibility, endosomal escape, innate immune activation, dosing frequency, tissue tropism, and access to infected organs. These challenges are especially important for systemic and neuroinvasive RVFV disease because large nucleic acid therapeutics typically require engineered delivery systems and have limited passive BBB penetration [75,76]. Further development should therefore prioritize infectious-virus validation of optimized N–RNA disruptors and delivery platforms that enable organ-targeted, systemic, or CNS-directed RNA-based therapy. This RNP-focused strategy provides a logical bridge to direct-acting approaches targeting the RVFV L protein and its enzymatic functions in viral RNA synthesis.

#### 5.1.4. Nucleoside Analogue Inhibitors

##### Ribavirin: Historical Benchmark with Delivery Constraints and Neurological Limitations

Ribavirin remains a historical RVFV comparator but has limited translational value. Its proposed activities include inhibition of viral RNA synthesis, depletion or perturbation of intracellular nucleotide pools through inosine monophosphate dehydrogenase inhibition, interference with viral mRNA capping, immunomodulatory effects, and lethal mutagenesis [77]. Efficacy is limited by toxicity, hemolytic anemia, teratogenicity concerns, modest potency, and inconsistent in vivo protection [21,78]. Nevertheless, ribavirin studies established two durable principles. Liposome-encapsulated ribavirin increased hepatic drug levels approximately five-fold and protected mice at doses ineffective as free ribavirin, highlighting the value of tissue-targeted delivery [19]. In addition, ribavirin combined with the interferon inducer poly(ICLC) remained effective when treatment began 48 h after infection, whereas either agent alone was ineffective at the same doses [20]. Critically, animals protected from peracute disease later developed delayed-onset neurologic disease with high brain viral burden [21], demonstrating that systemic survival does not ensure prevention of CNS seeding and/or encephalitis. RVFV antiviral studies should therefore pair conventional endpoints with brain viral load, neurologic scoring, neuropathology, and CNS pharmacology.

##### Favipiravir: Systemic Efficacy, Resistance Considerations, and Partial CNS Relevance

Favipiravir (T-705) is a benchmark nucleoside analog for RVFV. After conversion to favipiravir-ribofuranosyl-5′-triphosphate, it is incorporated by viral RNA-dependent RNA polymerases, inhibiting RNA synthesis and promoting lethal mutagenesis with characteristic G-to-A and C-to-U transitions [21,79,80,81]. Selection of partially favipiravir-resistant RVFV variants showed reduced fitness and attenuation [81]. Favipiravir has shown reproducible anti-RVFV activity across several strains and cell systems [21,24,81]. Favipiravir has also demonstrated protective efficacy in animal models of pathogenic RVFV infection. In the ZH501 hamster model, oral favipiravir at 200 mg/kg/day prevented mortality in at least 60% of animals when treatment began 1 or 6 h after infection and reduced serum and tissue viral loads [21]. In an inhalational ZH501 rat model, Caroline et al. reported that 66 of 72 favipiravir-treated Wistar-Furth rats survived lethal aerosol challenge, with protection maintained even when treatment was delayed up to 48 h post-infection [82]. However, protection remains strongest with early treatment, and efficacy against established encephalitis is unresolved. Future studies should relate plasma, liver, and brain exposure of favipiravir and its active triphosphate to brain viral burden and neurologic outcomes.

##### Galidesivir: Broad-Spectrum Nucleoside Analog

Galidesivir (BCX4430) is an adenosine analog that inhibits viral RNA synthesis after intracellular active triphosphate conversion and incorporation by viral RNA-dependent RNA polymerases as a non-obligate chain terminator [83]. RVFV studies showed improved survival in lethal mouse challenge [83] and protection of ZH501-challenged hamsters [22]. In hamsters, intramuscular and intraperitoneal treatment improved survival, reduced systemic viral replication, and markedly lowered infectious viruses in serum, liver, brain, and other tissues; the most protective regimen achieved 70% survival and markedly reduced tissue viral titers [22]. Compared with ribavirin in the same model, galidesivir produced better survival and tissue viral control study [22]. It therefore merits further development as a systemic polymerase-targeting candidate, but its delayed-treatment window, CNS exposure, and efficacy against neuroinvasive disease remain undefined.

##### 4′-Fluorouridine: Orally Active Broad-Spectrum Candidate

4′-fluorouridine (4′-FlU; EIDD-2749) is an orally active uridine-related ribonucleoside along with broad activity against RVFV and other bunyaviruses. After triphosphate conversion, it is expected to inhibit viral RNA synthesis [84]. 4′-FlU exhibits low-micromolar antiviral activity against RVFV and broad-spectrum activity across multiple bunyaviruses [23]. In BALB/c mice, once-daily oral therapy provided strong protection, reduced systemic and tissue viral loads, and retained post-exposure efficacy when initiated up to 3 days post-infection, although protection declined with treatment delay [23]. Its oral activity and delayed-treatment potential are notable, but neuroinvasive models should define brain exposure, CNS efficacy, neuropathology, and plasma–brain PK/PD relationships.

#### 5.1.5. Direct Targeting of the RVFV L Protein: Endonuclease Inhibition, Polymerase Inhibition, and Resistance

Recent advances in polymerase structure biology, endonuclease and minigenome-based functional assays have made the RVFV L protein increasingly tractable for structure-guided antiviral discovery. Kirby and colleagues identified MBXC-4522 through a FRET-based screen targeting the RVFV endonuclease. The compound inhibited endonuclease activity, stabilized the endonuclease domain, reduced RVFV MP-12-GFP reporter signal and plaque formation, and decreased viral RNA levels during ZH501 infection [25]. These findings validate endonuclease as druggable, although cytotoxicity necessitates medicinal chemistry optimization. Kral and colleagues integrated live-virus inhibition, minigenome validation, resistance selection, sequencing, and structural interpretation using a 3.5 Å cryo-EM structure of apo RVFV L protein [26]. Nucleoside analogs such as 2′-fluoro-2′-deoxycytidine and EIDD-1931 inhibited RVFV replication at low-micromolar concentrations and reduced RVFV minigenome activity, consistent with disruption of polymerase-dependent viral RNA synthesis [26]. This workflow distinguishes on-target polymerase inhibition from nonspecific antiviral effects and enables early assessment of resistance, cross-resistance, and fitness costs. Future L-directed programs should prioritize potency, resistance barriers, pharmacokinetics, delayed-treatment activity and efficacy in neuroinvasive disease models.

#### 5.1.6. Targeting NSs-Mediated Virulence Functions

NSs is the major RVFV virulence factor and an attractive antiviral target because it suppresses host antiviral defenses through multiple mechanisms, including inhibition of host transcription, suppression of IFN-β induction, degradation of PKR, disruption of TFIIH-associated transcriptional machinery, and formation of nuclear filamentous structures [85,86,87,88,89]. The attenuation of ΔNSs viruses in vaccine and pathogenesis studies further supports the importance of NSs in RVFV virulence, although genetic attenuation alone does not establish NSs as a validated direct small-molecule antiviral target [90]. Compared with strategies targeting the L protein, viral fusion, or N–RNA interactions, direct pharmacologic targeting of NSs remains less developed, likely because NSs acts mainly through host-modulatory and protein-interaction-based mechanisms rather than a well-defined catalytic activity. Therefore, NSs-directed discovery will likely require cell-based and mechanism-based screening approaches. Several studies suggest that NSs-associated functions can nevertheless be therapeutically targeted. RNAi targeting the S segment reduced NSs expression and decreased NSs-associated pathogenic effects in cultured cells [71]. Bortezomib inhibited RVFV multiplication in human cells, disrupted NSs nuclear filament formation, altered NSs-associated host interactions, and increased IFN-β expression [65]. MLN4924 blocked NSs-dependent PKR degradation by interfering with the SCF^FBXW11^–NSs E3 ligase pathway, thereby restoring PKR activation and reducing RVFV protein synthesis [91]. This pathway is further supported by evidence that NSs recruits FBXW11 and β-TRCP1 to promote PKR degradation [92]. However, these compounds should be considered NSs-function-associated or host-pathway-directed inhibitors rather than confirmed direct NSs-binding antivirals. Future NSs-directed antiviral discovery should prioritize assays that measure restoration of IFN-β induction, rescue of host transcription, preservation of PKR or TFIIH-p62, disruption of NSs nuclear filaments, and inhibition of NSs–host protein interactions. High-content imaging, IFN-β reporter assays, proteomics, reverse genetics, and resistance studies could help determine whether candidate compounds specifically disrupt NSs-associated functions rather than causing nonspecific host–cell toxicity. Overall, NSs remains a biologically compelling virulence target for RVFV therapeutic development; however, its translation into a direct antiviral strategy will require stronger assay development, target validation, selectivity profiling, resistance mapping, and in vivo efficacy testing.

#### 5.1.7. Screening-Derived Small Molecules and Late-Stage Inhibitors

Phenotypic, imaging-based, and reporter-virus platforms have expanded RVFV antiviral discovery by identifying inhibitors of viral replication, assembly, and egress. Mudhasani and colleagues screened 849 protease-inhibitor-library compounds and advanced four hits for mechanistic analysis: C795-0925 and F694-1532 inhibited viral replication, whereas D011-2120 and G202-0362 impaired virus egress by disrupting microtubule organization, Golgi trafficking, or trans-Golgi budding [31]. These findings illustrate how high-content screening can move beyond inhibition identification to assign life-cycle stage and reveal potential host–cell liabilities. MP12 reporter systems have similarly enabled lower-containment antiviral discovery. Lang and colleagues identified 6-azauridine and mitoxantrone as inhibitors of MP-12. In STAT-1 knockout mice, mitoxantrone delayed disease onset and reduced severity, whereas 6-azauridine was less protective [32]. These results support MP-12-based screening but require confirmation with virulent RVFV strains and models that preserve relevant immune, tissue-tropism, and neuroinvasion features. Repurposing and combination studies further emphasize the need to align antiviral potency with exposure. Favipiravir plus nitazoxanide showed additive in vitro activity against several Bunyaviricetes, including RVFV, and nitazoxanide appeared to affect late replication or progeny-virus production [24]. However, limited mouse exposure and lack of robust standalone protection indicate that nitazoxanide should be viewed as a late-stage antiviral candidate and combination partner, not a validated RVFV monotherapy [24]. Overall, screening-derived candidates should therefore be prioritized not only by potency and cytotoxicity indices but also by confirmation of the pathogenic virus, assessment of resistance barriers, tissue-exposure profiling, and efficacy testing in delayed-treatment and neuroinvasive disease models.

### 5.2. Host-Directed Antiviral Strategies

Host-directed antivirals complement direct-acting agents by targeting cellular pathways that RVFV exploits for replication, immune evasion, dissemination, and virus-induced tissue injury. These strategies may raise the barrier to viral escape and retain activity across diverse RVFV strains; however, their value depends on separating antiviral benefit from toxicity, immunosuppression, or disruption of essential host functions. This balance is especially important in neuroinvasive disease, where host-directed therapies must suppress viral replication without weakening protective immunity or worsening inflammation in the CNS.

#### 5.2.1. NF-κB/IKK Signaling and Curcumin

RVFV infection remodels host signaling networks that may be therapeutically exploitable. Narayanan and colleagues demonstrated that MP-12 infection activates NF-κB signaling and induces the formation of a low-molecular-weight, IKK-β–containing complex, termed IKK-2, that retains kinase activity. Curcumin inhibited IKK-2 kinase activity, decreased extracellular infectious virus production, altered NSs phosphorylation and cell-cycle progression, and suppressed virulent ZH501 replication in vitro and hepatic replication in MP12-infected animals [63]. However, curcumin’s poor bioavailability, pleiotropy, and uncertain target specificity limit its translational relevance [93,94]. The main importance of this study is its mechanistic insight. It identifies NF-κB/IKK signaling, NSs phosphorylation, and infection-specific host protein complexes as potential targets for developing more selective antiviral inhibitors.

#### 5.2.2. Ubiquitin-Proteasome System and Bortezomib

The ubiquitin-proteasome system supports NSs-associated degradation of antiviral host factors and disruption of innate immune signaling. Keck and colleagues demonstrated that the FDA-approved proteasome inhibitor bortezomib markedly reduced both intracellular and extracellular RVFV levels. Bortezomib also disrupted the formation of NSs nuclear filaments, modified NSs interactions with the host proteins SAP30 and mSin3A, altered host-protein ubiquitination, and enhanced IFN-β expression [65]. Although these findings implicate proteasome-dependent NSs–host interactions as antiviral targets, global proteasome inhibition is unlikely to be acceptable for acute RVFV infection because of its potential toxicity. Future efforts should therefore focus on selective disruption of NSs–host interactions or NSs-mediated degradation of antiviral factors rather than broad proteasome blockade.

#### 5.2.3. Protein Phosphatase-1 and 1E7-03

Protein phosphatase-1 (PP1) is another host factor required for efficient RVFV replication. Baer and colleagues showed that PP1α knockdown or treatment with the PP1-targeting compound 1E7-03 reduced viral titers across multiple cell lines, likely by suppressing viral RNA production and disrupting interactions among viral RNA, L polymerase, and nucleoprotein [66]. PP1-regulated phosphorylation networks may therefore provide higher-resistance-barrier host targets, but PP1’s broad cellular functions make selectivity the key challenge. Defining RVFV-relevant PP1 holoenzymes, regulatory subunits, and virus-induced phosphatase interactions will be essential for pathway-selective intervention.

#### 5.2.4. mTOR/p70S6K, MAPK Signaling and Kinase Inhibitors

Host translational-control pathways are promising although their therapeutic use may be limited by toxicity. RVFV infection activates mTOR-linked phosphorylation of p70 S6 kinase, S6 ribosomal protein, and eIF4G. Rapamycin reduced viral titers, decreased p70 S6K phosphorylation, increased survival, and mitigated clinical disease in RVFV-challenged mice [67]. Subsequent studies showed that p70 S6K inhibition reduced RVFV titers, whereas coordinated inhibition of p70 S6K with MAPK-associated signaling produced substantially stronger suppression, including synergy between rapamycin with p38 MAPK or ERK inhibitors [68]. These results suggest that RVFV can tolerate partial blockade of single signaling branches but is more vulnerable to coordinated disruption of parallel translation-supportive pathways. Translational development should prioritize combinations that suppress RVFV replication without unacceptable immunosuppression, metabolic toxicity, or impairment of tissue repair, particularly in severe hepatic or neuroinvasive disease.

#### 5.2.5. Innate Immune Stimulation: Poly(ICLC), FMDV-Derived RNAs, and Tilorone

Because RVFV is highly sensitive to type I interferon, making innate immune stimulation an attractive but strongly timing dependent. Poly(ICLC), a stabilized double-stranded RNA interferon inducer, protected animals after repeated post-infection dosing and showed improved efficacy with ribavirin [20]. Synthetic noninfectious foot-and-mouth disease virus (FMDV)-derived RNA transcripts similarly induced systemic antiviral and pro-inflammatory cytokines and protected mice in a timing- and transcript-dependent manner [70]. Tilorone also inhibited MP-12 and virulent ZH501 in vitro and improved mouse survival when given immediately after lethal ZH501 challenge, with partial protection after one-day delayed prophylaxis [69]. These agents are therefore best viewed as prophylactic or very early post-exposure adjuncts that reduce peripheral replication before systemic dissemination or CNS seeding. Their translational use is constrained by a narrow therapeutic window, inflammatory toxicity, and the risk that delayed systemic immune activation could worsen tissue injury, especially in neuroinvasive disease. The neuron-focused Pam3CSK4 study further cautions that canonical interferon pathways may not efficiently protect all CNS-relevant cell types [28].

#### 5.2.6. Redox Modulation and NSC62914

Redox modulation remains an early host-directed strategy for RVFV. Panchal and colleagues identified NSC62914, an antioxidant small molecule with broad-spectrum antiviral activity against RVFV, Lassa virus, Venezuelan equine encephalitis virus and filoviruses. Mechanistic studies indicated reactive oxygen species scavenging and induction of oxidative-stress-induced genes, but other known antioxidants failed to reproduce the antiviral effect, suggesting additional or context-dependent mechanisms beyond nonspecific redox buffering [64]. Thus, antioxidant activity alone does not predict antiviral efficacy, and redox-active compounds may influence viral replication, cell viability, inflammatory signaling, and assay readouts through overlapping mechanisms. Redox-modulating antivirals remain at an early stage of RVFV development and are therefore best considered adjunctive agents that require rigorous target definition, structure–activity analysis, and validation in hepatic and neuroinvasive RVFV disease models, including assessment of brain exposure, glial responses, neuronal protection, and inflammatory neurotoxicity.

## 6. Combination Therapy

Combination therapy represents a biologically rational but still underdeveloped strategy for RVFV because severe disease spans early systemic replication, hepatic amplification, CNS seeding, and established neuroinvasive disease in some cases. Early studies showed that ribavirin plus poly(ICLC) improved post infection therapeutic efficacy, supporting combined direct antiviral activity and innate immune stimulation [20]. Favipiravir plus ribavirin improved survival and reduced titers relative to monotherapy in a delayed treatment hamster model, supporting the broader principle that nucleoside analog combinations may extend the therapeutic window or reduce tissue-associated viral burden [21]. Chaput and colleagues identified the combination favipiravir and nitazoxanide as having additive in vitro activity against several *Bunyaviricetes* viruses, including RVFV. In a severe murine RVFV model, this combination reduced viral replication and prolonged time to death but did not confer complete protection; pharmacokinetic analyses showed favorable favipiravir exposure but low nitazoxanide exposure in mice [24]. These findings suggest that combining antivirals with different mechanisms may broaden activity, reduce the risk of resistance, and target both early systemic infection and later neurologic disease. However, an effective combination must have compatible pharmacokinetics, nonoverlapping toxicities, and sufficient drug exposure in peripheral organs, such as the liver and spleen, as well as in the brain and other CNS tissues. Future combination studies should therefore move beyond survival and serum viral-load endpoints to incorporate delayed-treatment designs, resistance-barrier testing, plasma–liver–brain exposure, CNS seeding, and assessment of whether paired agents prevent or suppress established encephalitic disease.

## 7. A Unifying Framework for RVFV Neuropathogenesis and Therapeutic Implications

**Figure 4:**
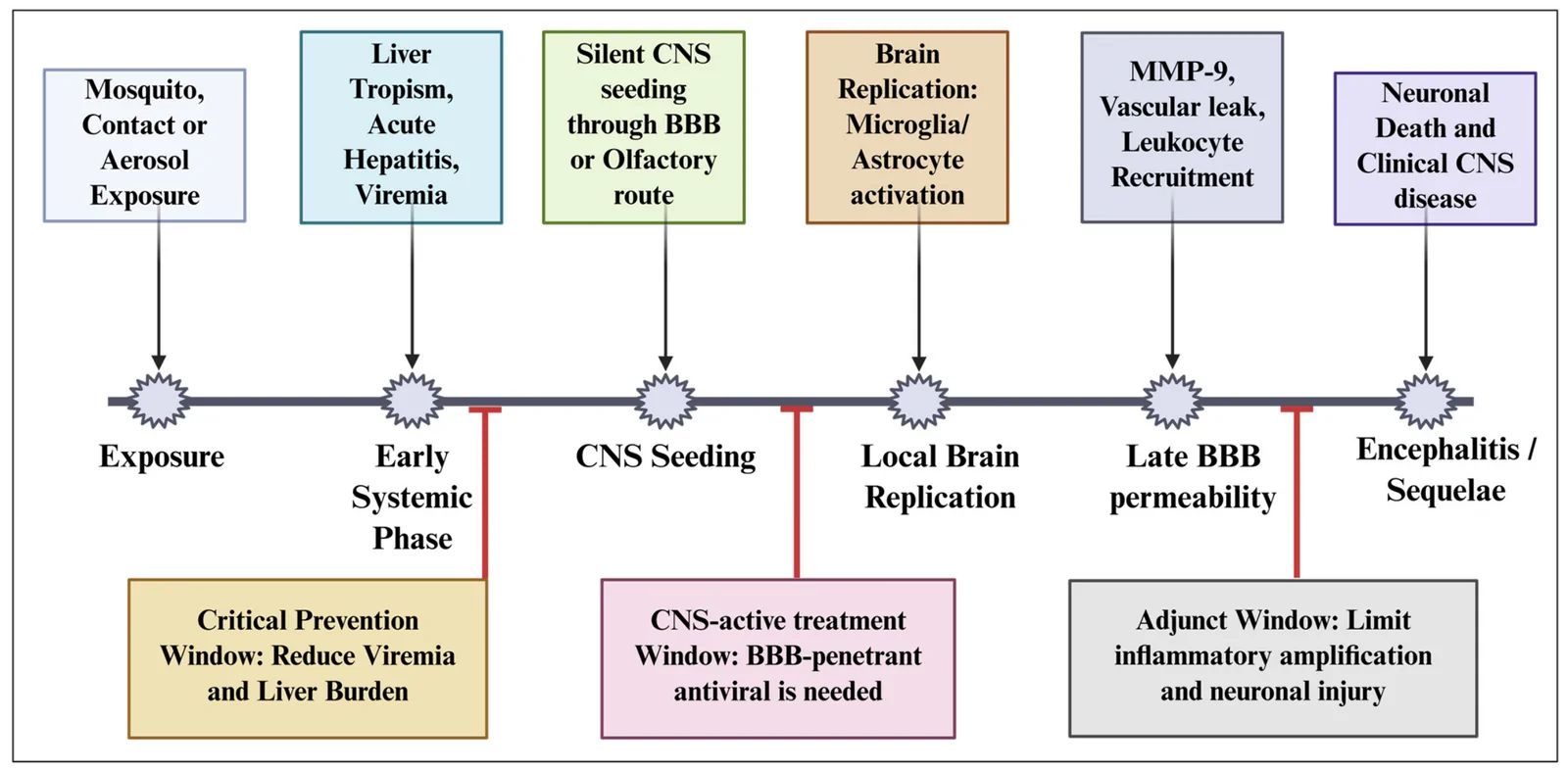
Temporal model of RVFV progression from acute systemic disease to delayed encephalitis. RVFV infection may progress from early systemic replication, viremia, and acute hepatitis to delayed CNS seeding through BBB/neurovascular or olfactory routes in infected humans. After CNS entry, local brain replication, glial activation, delayed BBB permeability, MMP-9-associated vascular leakage, leukocyte recruitment, and neuroinflammation may culminate in neuronal death and encephalitis. This model highlights three potential therapeutic windows: early antiviral treatment after clinical signs and RVFV infection are confirmed, typically 3–4 dpi, to reduce viremia and hepatic viral burden; CNS-active treatment with BBB-penetrant antivirals during the post-acute period, typically 1 to several weeks after recovery from systemic symptoms such as hemorrhagic fever, to limit CNS infection; and adjunctive therapy after neurological signs emerge to reduce inflammatory and neuronal injury. Created in BioRender. Roy, S. (2026) https://BioRender.com/.

Synthesis of the neuropathogenesis and antiviral literature supports a temporally structured model in which early systemic replication, hepatic amplification, subclinical CNS seeding, established parenchymal infection, and subsequent neuroinflammatory injury form overlapping therapeutic windows (Figure 4). During early viremia, RVFV may access CNS through exposure-specific routes, including olfactory-cribriform dissemination following inhalational exposure or BBB/neurovascular traversal after peripheral exposure. CNS seeding may initially remain clinically silent and precede detectable BBB permeability. Once RVFV replicates in brain tissue, local viral replication activates microglia and astrocytes to induce MAVS-dependent antiviral programs, interferon-stimulated genes, cytokines, and chemokines. If viral control fails, leukocyte recruitment, MMP-9-associated BBB permeability, inflammatory cell-death pathways, and direct viral cytopathology converge to drive neuronal injury, encephalitis, and persistent neurological sequelae.

This framework accommodates both respiratory and mosquito-borne RVFV disease without invoking a single universal CNS entry route and explains why encephalitis can emerge after apparent resolution of systemic illness. Neurologic disease may reflect progression from limited CNS seeding during viremia to local viral replication, brain-intrinsic immune activation, leukocyte recruitment, and neurovascular dysfunction that together produce clinically evident disease.

This model has direct implications for antiviral and vaccine development. The first therapeutic window occurs during systemic infection, when rapid suppression of hepatic replication and viremia may reduce CNS seeding, consistent with the protective role of CD4-dependent antibody responses and the sufficiency of humoral immunity in the CC057/Unc model [50,55]. A second therapeutic window may emerge after subclinical CNS entry, when treatment efficacy is likely influenced by brain exposure, timing of therapy, antiviral activity in CNS-resident cells, and the extent of established neuropathology. Antivirals that reduce peripheral viral burden may protect against acute hepatitis, but their ability to prevent delayed encephalitis should be evaluated using CNS-relevant endpoints, including brain drug exposure, CNS viral burden, neurologic outcomes, and neuroinvasive disease models. This issue is particularly relevant to post-exposure treatment, laboratory accidents, high-risk occupational exposures, and patients with early neurological signs. A third window involves limiting inflammatory and neurodegenerative injury after CNS replication has initiated glial activation, leukocyte influx, BBB permeability, and neuronal cell death. At this stage, direct antiviral therapy alone may be insufficient, and adjunctive strategies targeting modulation of MMP activity, vascular leakage, cytokine amplification, or regulated neuronal cell death pathways need to be evaluated. Because MAVS-dependent microglial signaling and adaptive immunity contribute to host protection; however, nonspecific immunosuppression could compromise viral control. Combination regimens should therefore prioritize rapid viral suppression followed by targeted neuroprotective or anti-inflammatory interventions that preserve protective immunity while limiting CNS injury.

## 8. Conclusions and Future Directions

RVFV neuropathogenesis is best understood as a route-dependent, multistage process in which early systemic replication and hepatic amplification may progress to silent CNS seeding, parenchymal infection, neuroinflammatory injury, and long-term neurologic or ocular sequelae. However, key gaps remain in defining how exposure route, viremia, BBB-associated infection, leukocyte trafficking, and brain-intrinsic immunity regulate CNS invasion, and whether BBB disruption initiates neuroinvasion or mainly amplifies established CNS injury. New percutaneous encephalitis models should therefore be used to identify the genetic and immune factors that drive progression from acute hepatitis to delayed encephalitis, while distinguishing direct viral neuronal injury from secondary neuroinflammatory damage [10,11,51]. These models should also be incorporated into antiviral testing, because reducing peripheral viremia or liver disease may not be sufficient to prevent CNS seeding or delayed brain infection [11,21,82]. These uncertainties have direct therapeutic implications.

Future RVFV countermeasure development should be prioritized based on feasibility, CNS relevance, and outbreak preparedness. In the near term, existing antiviral pipelines should incorporate CNS-focused evaluation, including BBB-permeability prediction, efflux-transporter profiling, plasma–liver–brain pharmacokinetics, CNS PK/PD assessment, delayed-treatment testing, brain viral-burden quantification, neurologic scoring, and neuropathologic assessment in neuroinvasive animal models [95,96]. These endpoints are particularly important for small-molecule direct-acting antivirals, which are likely the most practical option during RVFV outbreaks because they can be manufactured, scaled, and deployed more easily than complex delivery systems. Another near-term priority is to evaluate combination therapies that target distinct stages of disease. For instance, systemic neutralizing antibodies or antibody cocktails may reduce early viremia and limit CNS seeding, whereas small molecules with adequate brain exposure may help control established brain infection. These combinations should be tested in models that separately assess protection against acute hepatic lethality, delayed encephalitis, and neuro-ocular disease. In contrast, advanced CNS-delivery approaches, including receptor- or transporter-mediated BBB shuttles, nanocarriers, exosomes, intranasal delivery, and focused ultrasound-assisted BBB opening, should currently be considered exploratory. Although these strategies may improve CNS delivery of antivirals, antibody fragments, or biologics, they require careful validation of timing, biodistribution, safety, and neuroinflammatory risk in acute RVFV infection models [95,96,97,98]. Recent progress with transferrin-receptor-mediated BBB-crossing biologics supports the feasibility of receptor-based CNS delivery, but its application to acute viral infection remains unproven and should be evaluated cautiously [99]. AI-guided screening and BBB-permeability prediction may further help prioritize CNS-active candidates, but these tools should complement, not replace experimental validation in BBB models, CNS-cell antiviral assays, CNS PK/PD studies, and neuroinvasive animal models [100]. Overall, future RVFV therapeutic development should first prioritize outbreak-ready antivirals with defined systemic and CNS exposure, while longer-term delivery technologies may provide additional options for established or high-risk neuroinvasive disease.

## Figures and Tables

**Table 1:** Evidence for RVFV neuroinvasion and neuropathogenesis mechanisms.

Mechanistic Domain	Key Findings	Model	Virus Strain	Proposed Role in RVFV Neuropathogenesis
Olfactory/cribriform neuroinvasion	Detected RVFV in the olfactory epithelium, cribriform plate, and olfactory bulb.	Aerosol rat model	ZH501	Serves as the dominant neuroinvasion route after inhalational or mucosal exposure [45]
Aerosol exposure disease severity	Linked aerosol exposure to earlier and more severe neuropathology than subcutaneous infection.	Aerosol or subcutaneous mouse model	ZH501	Accelerates CNS involvement and shortens the therapeutic window after aerosol exposure [46]
Peripheral/percutaneous neuroinvasion	Demonstrated that footpad infection of CC057/Unc mice caused self-limited liver disease followed by delayed neurologic signs, CNS viral RNA, brain lesions, and viral antigen in the brain.	CC057/Unc mouse	ZH501	Provides a mosquito-bite-like exposure model to study delayed CNS disease after peripheral RVFV infection [40]
Direct BBB crossing	Demonstrated endothelial infection and apical-to-basal crossing without overt barrier disruption.	In vitro human BBB model	MRU25010-30; Mayotte 2008/00099	Supports hematogenous CNS entry through BBB-associated cells [47]
BBB permeability	Associated late vascular leakage with CNS replication and increased MMP-9.	Aerosol rat model	ZH501	Amplifies established encephalitis [48]
Leukocyte-associated “Trojan horse” entry	Observed myeloid-cell CNS infiltration, although leukocyte-mediated viral entry remained unproven.	Aerosol or subcutaneous rat model	ZH501	Provides a possible accessory route during inflammation [49]
Adaptive immune control	Showed that B cells and CD4 T cells promoted viral clearance, whereas CD4 depletion increased neurological disease.	Subcutaneous, intranasal or footpad mouse model	ZH501-derived ΔNSs; ΔNSm-ΔNSs recombinant RVFV	Limits systemic viral replication and reduces the risk of CNS disease [50]
Microglial innate immunity	Linked MAVS deficiency to increased brain viral burden, mortality, and impaired microglial responses.	Intranasal mouse model	ZH501; MP-12	Restricts RVFV replication through MAVS-dependent type I interferon and ISG responses in microglia [51]
Myeloid inflammation	Observed inflammatory neutrophil and macrophage influx, although neutrophil blockade did not improve outcomes.	Aerosol or subcutaneous rat model	ZH501	Contributes to inflammatory encephalitis [49]
Brain-intrinsic injury	Confirmed RVFV replication, neuronal infection, glial activation, chemokine induction, and apoptosis in brain slices.	Ex vivo rat brain slice culture	ZH501	Supports local CNS replication and virus-induced tissue injury [52]
Neuronal cell death	Detected apoptosis, pyroptosis, and necroptosis markers in brain tissue and primary neurons.	Aerosol rat model; primary neuronal cultures	ZH501; RVFV-delNSs/NSm	May drive lethality and long-term neurologic sequelae [53]

**Table 2:** Antiviral agents and therapeutic strategies evaluated against Rift Valley fever virus.

Antivirals	Primary Target or Pathway	In Vivo Verification	Main Evidence/Model	Key Finding(s)
Liposome-encapsulated Ribavirin	Liver-targeted delivery; RdRp/nucleotide metabolism	Yes	Zagazig 501 strain (ZH501); Female Swiss Webster mice	Increased hepatic exposure and efficacy at lower doses [19]
Poly(ICLC) & Ribavirin	Innate induction; RdRp/nucleotide metabolism	Yes	ZH501; Female Swiss Webster mice	Remained effective when administered up to 48 h post-infection [20]
Favipiravir	RdRp inhibition; Lethal mutagenesis	Yes	ZH501; Female Golden Syrian hamster	Protected from peracute disease and reduced delayed neurologic disease [21]
Galidesivir	RdRp/Adenosine analog	Yes	ZH501; Female Golden Syrian hamster	Improved survival and reduced infectious virus in tissues [22]
4′-FlU	RdRp/Ribonucleoside analog	Yes	ZH501; Male & Female BALB/c mice	Showed low-micromolar potency and oral protective efficacy [23]
Favipiravir & Nitazoxanide	RdRp mutagenesis; Late replication; Release effects	Yes	Chad 2001; C57BL/6 mice	Produced additive antiviral effects, reduced viral replication, and prolonged survival [24]
MBXC-4522	RVFV endonuclease	No	MP12 & ZH501; Invitro FRET assay	Inhibited endonuclease activity through a metal-independent mechanism [25]
Polymerase inhibitor panel	RdRp/RVFV L protein	No	ZH548; minigenome, resistance selection, cryo-EM	Identified resistance mutations and mapped them structurally [26]
Gc fusion peptide	Gc-mediated fusion	No	ZH548 and other viruses; in vitro assays	Blocked viral fusion and demonstrated broad-spectrum antiviral activity [27]
Pam3CSK4	Fusion inhibition, TLR2-independent activity	Yes	MP-12 in primary rodent cortical neurons; ZH501 in female C57BL/6 mice using an intracranial encephalitis model	Protected from brain infection and mortality [28]
N-RNA HTS hits	N-RNA binding	No	Fluorescence polarization-based HTS screen of 26,424 compounds	Established a screening platform for identifying N–RNA interaction inhibitors [29]
Suramin	N-RNA binding; Cellular entry/uptake	No	MP12; Human cell culture; biochemical assays	Disrupted ribonucleoprotein complex formation and inhibited viral replication [30]
C795-0925	Viral replication	No	MP12 & ZH501; High-content screening	Inhibited RVFV replication with minimal effects on cellular morphology [31]
D011-2120	Microtubules dynamic/Golgi-mediated egress	No	MP12 & ZH501; High-content screening	Blocked microtubule polymerization and impaired viral egress [31]
G202-0362	Trans-Golgi mediated budding/egress	No	MP12 & ZH501; High-content screening	Blocked viral budding with minimal effects on cellular morphology [31]
Mitoxantrone	Likely viral replication/assembly effects	Yes	MP-12; in vitro screening; STAT1-knockout mice	Delayed disease onset and reduced disease severity [32]
6-azauridine	Nucleotide metabolism	Yes	MP-12; in vitro screening; STAT1-knockout mice	Inhibited MP-12 replication in vitro [32]
CNX-1351	PI3K-Akt-associated post-entry replication	No	in vitro single-cycle viral replicon particle (MP12) screening	Identified as a broad RNA virus antiviral candidate [62]
Curcumin	NF-kappaB/IKK-2; NSs phosphorylation	Yes/Hepatic viral-load readout	MP-12 and ZH501; INFAR^-/-^mice;	Reduced RVFV replication [63]
NSC62914	Redox and ROS signaling	No	MP12; invitro assay	Inhibited RVFV and other viruses [64]
Bortezomib	Proteasome dependent NSs-host interactions	No	MP12; invitro assay	Reduced viral loads and disrupted NSs filaments [65]
1E7-03	Protein phosphatase-1	No	MP12; in vitro assay	Reduced viral titers and viral RNA production [66]
Rapamycin	mTOR/p70S6K translation signaling	Yes	ZH501; BALB/c	Reduced viral titers and improved survival [67]
PF-4708671 combinations	p70S6K, p90RSK, MAPK mediated translation control	No	MP12; in vitro assay	Robustly suppressed viral replication through combined kinase inhibition [68]
Tilorone	Innate, lysosomal, and glycoprotein-associated effects	Yes	ZH501; BALB/c mice	Achieved 80% survival when treatment was initiated immediately [69]
FMDV NCR RNAs	PRR activation; type I IFN induction	Yes	Strain 56/74; BALB/c mice	Protected mice in a timing-dependent manner [70]
N/NSs shRNAs	RNA interference-mediated gene silencing	No	1981 South Africa isolate; in vitro assay	Reduced viral replication through N-targeting shRNAs and reduced cytotoxicity through NSs-targeting shRNAs [71]
N siRNAs	RNA interference-mediated gene silencing	No	MP12; in vitro assay	Suppressed viral protein expression and preserved PKR expression [72]
D2 siRNA	N mRNA nucleotides 488-506	No	Egyptian cell culture adapted RVFV strain; in vitro assays	Strongly inhibited infection when administered before or after transfection [73]

## Data Availability

All original contributions generated for this review are included in the article.

## Abbreviations

The following abbreviations are used in the manuscript.

RVFV	Rift Valley Fever Virus
WHO	World Health Organization
DC-SIGN	Dendritic cell-specific intercellular adhesion molecule-3-grabbing non-integrin
L-SIGN	Liver/lymph node-specific intercellular adhesion molecule-3-grabbing non-integrin
LRP1	Low-density lipoprotein receptor-related protein 1
BBB	Blood–brain barrier
hBLEC	Human Brain-like endothelial cells
CNS	Central nervous system
NSs	Nonstructural protein encoded by the RVFV S segment
MAVS	Mitochondrial antiviral-signaling protein
CCR5	C–C chemokine receptor type 5
RNA	Ribonucleic acid
HIV	Human immunodeficiency virus
SARS-CoV-2	Severe acute respiratory syndrome coronavirus 2
HBV	Hepatitis B virus
HDV	Hepatitis D virus
TLR2	Toll-like receptor 2
TLR7	Toll-like receptor 7
shRNA	Short hairpin RNA
siRNA	Small interfering RNA
RNP	Ribonucleoprotein
STAT1	Signal transducer and activator of transcription
PK/PD	pharmacokinetics/pharmacodynamics
